# Clinical gait analysis use in management of children with cerebral palsy: Qualitative study

**DOI:** 10.1111/dmcn.70216

**Published:** 2026-02-21

**Authors:** Marie Lévesque‐Jalbert, Katia Turcot, Marie Rousseau‐Demers, Yosra Cherni, Philippe C. Dixon, Laurent Ballaz, Louis‐Nicolas Veilleux, Maxime T. Robert

**Affiliations:** ^1^ Département de Kinésiologie, Faculté de Médicine Université Laval Québec Canada; ^2^ Centre interdisciplinaire de recherche en réadaptation et intégration sociale (Cirris) Québec Canada; ^3^ Centre de Recherche Azrieli du CHU Ste Justine Montréal Canada; ^4^ École de kinésiologie et sciences de l'activité physique, Faculté de Médecine Université de Montréal Montréal Canada; ^5^ Department of Kinesiology and Physical Education McGill University Montreal Canada; ^6^ Département des sciences de l'activité physique Université du Québec à Montréal—UQAM Montréal Canada; ^7^ Motion Analysis Center Shriners Hospital for Children Montreal Canada; ^8^ Division of Surgical and Interventional Sciences, Department of Surgery McGill University Montreal Canada; ^9^ École des sciences de la réadaptation, Faculté de médecine Université Laval Québec Canada

## Abstract

**Aim:**

To identify the barriers and facilitators that shape how clinical gait analysis (CGA) informs family decision‐making in different contexts.

**Method:**

A qualitative descriptive study design was chosen to explore the current clinical landscape. Semistructured interviews were conducted with 15 healthcare professionals and eight caregivers (*n* = 23) from three pediatric rehabilitation centers in Quebec, Canada, that provide CGA to children with cerebral palsy. Data were analyzed thematically with an inductive approach using NVivo software.

**Results:**

Key barriers include a lack of structure, limited financial and human resources, and restricted access to CGA. The primary facilitators are healthcare professionals and caregivers who aim to enhance the care process by more effectively integrating CGA. Other common topics include the benefits of care management with CGA and the need to clarify the expertise required to ensure the optimal functioning and clinical application of a gait laboratory.

**Interpretation:**

Participants emphasized the importance of addressing disparities, securing institutional support, and promoting intercenter collaboration to achieve sustainable improvements in the care of ambulatory children with cerebral palsy.

## Graphical abstract

Barriers and facilitators related to the use of clinical gait analysis (CGA), as identified by healthcare professionals working with children with cerebral palsy and by caregivers. The colored triangles represent the different barriers reported at various stages along the CGA care pathway. Facilitators were identified by both caregivers and healthcare professionals, emphasizing the importance of interdisciplinary collaboration in supporting the effective use of CGA.

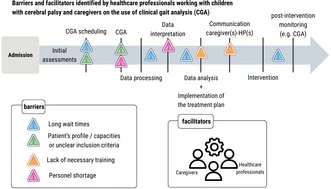

**Abbreviations**
CGA: clinical gait analysis

**What this paper adds**

- Healthcare professionals want to improve interdisciplinary collaboration by introducing a unified framework.
- The lack of human and financial resources limits access to clinical gait analysis (CGA).
- Healthcare professionals want to develop intercenter partnerships.
- Guidance is needed to identify key knowledge for interpreting CGA.

Effective management of neuromotor disorders affecting ambulatory function requires the development of organized and comprehensive treatment plans, with particular attention to the patient's profile and goals.^1^ For example, children with cerebral palsy (CP) present a vast range of functional levels and sensorimotor deficits.^2^ This leads to complex ambulatory profiles requiring consideration of numerous treatment options, significantly influencing decision‐making.^3^ Clinical assessments, such as the Gross Motor Function Measure, provide a measure of changes in gross motor function over time or after intervention.^4^, ^5^ However, the Gross Motor Function Measure is based on observations and does not provide an in‐depth gait analysis. The use of clinical gait analysis (CGA) can be a valuable tool to compensate for the limitations of clinical tests in establishing functional status.

In the last decades, CGA has gained popularity among healthcare professionals as an assessment tool.^6^, ^7^, ^8^ Combined with observational and clinical evaluations, CGA offers a comprehensive understanding of gait‐related issues and helps determine relevant treatment options through objective analysis.^6^, ^9^ Specifically, CGA characterizes walking difficulties by identifying which biomechanical variables differ from the norm.^10^

A case study highlighted the importance of using multiple reliable and clinically relevant measures when evaluating treatment outcomes.^11^ For example, CGA helps quantify key gait parameters, such as knee range of motion, hip rotation, and foot progression angle, which are identified as influencing the success of complex interventions, including single‐event multilevel surgeries.^12^ Consequently, CGA objectivity can impact the implied interdisciplinary nature of the decision‐making process by increasing confidence and consensus among healthcare professionals and ensuring that caregivers have a clearer understanding of the treatment plan.^13^, ^14^

Despite its positive impact on the care of children with CP, implementing CGA within clinical settings remains challenging because of multiple barriers. Notably, there is no one‐size‐fits‐all approach to treatment, primarily because of the population's heterogeneity.^6^, ^15^ Additionally, CGA involves a multidimensional structure that requires specific equipment and knowledge. This necessitates the involvement of several healthcare professionals and experts from various contiguous disciplines, each with distinct backgrounds and needs.^14^ Recent initiatives have highlighted the diversity of national policies and clinical practices, which may hinder future efforts to establish guidelines for CGA across Europe.^7^, ^16^ This study aims to identify the barriers and facilitators related to healthcare professionals working with children with CP and understand how CGA impacts families' decision‐making processes in various contexts.

## METHOD

### Study design

A descriptive qualitative multicenter study was conducted using semistructured interviews to explore perceptions, experiences, and contextual factors related to the use of CGA in pediatric rehabilitation. Given the limited documentation of both healthcare professionals and caregivers' perspectives combined, an interpretive qualitative approach was adopted to capture the meanings participants attribute to their experiences. The study was grounded in a constructivist paradigm, recognizing that participants' knowledge is actively constructed by their experiences and environment. Thus, the meanings are co‐constructed through interaction between participants and researchers. An inductive thematic analysis was used to identify patterns of meaning across participants, moving beyond surface descriptions to interpret how experiences of CGA are understood and negotiated across contexts and roles.

The Centre intégré universitaire de santé et de services sociaux de la Capitale‐Nationale Research Ethics Committee (2023–2629), as well as the local ethics committee from the two other sites, approved the research protocol, and all participants provided written informed consent. The Consolidated Criteria for Reporting Qualitative Research (COREQ) checklist was used to report this study design, ensuring rigor.

### Participants and recruitment

A total of 23 participants were recruited (15 healthcare professionals and eight caregivers of children with CP) using a purposive sampling with a maximum variation strategy. This strategy was selected to capture a range of professional disciplines' experiences as well as caregivers' and their adaptations to their own conditions across centers.^17^ Recruitment stopped when sufficient redundancy of information was achieved across participant groups.

Participants were recruited from all three centers. The MSc research trainee (MLJ) assigned to the project first contacted potential participants via email.

Each center was asked to recruit at least one or two caregivers and one healthcare professional from each discipline. To be included in this study, healthcare professionals were required to work with children or adolescents with CP and be familiar with CGA, regardless of their level of experience. Caregivers need to have witnessed at least one CGA. Participants had to be able to communicate either in French or English. No prior collaborative relationship was established between the research trainee conducting the interviews (MLJ) and the participants.

### Participating centers

The centers are the three main CGA centers in the province of Quebec: Shriners Hospital for Children from Montreal, Institut de Réadaptation en Déficience Physique de Québec from the Centre interdisciplinaire de recherche en réadaptation et intégration sociale, and Centre de Réadaptation Marie Enfant—Centre Hospitalier Universitaire Sainte‐Justine.

There are known disparities in resources across centers. One has a laboratory coordinator and a designated gait laboratory team in a private setting. Another collaborates with a research gait laboratory and relies mainly on hospital foundation funding. The third relies solely on research grants. The interviewer (MLJ) visited and observed CGA sessions in each center. Research team members attend interdisciplinary meetings for all three centers.

### Procedure

The multidisciplinary research team developed initial interview guides in French. Then, two team members tailored the guides to the two participant categories: healthcare professionals and caregivers (Appendix S1 and Appendix S2). Each guide was adapted mainly by removing technical questions from the caregivers' guide. The sociodemographic section included eight questions for healthcare professionals (e.g. years of experience working with children with CP) and 13 for caregivers (e.g. number of CGAs completed), including age, sex, occupation, and education level for all participants. The open‐ended sections included 18 questions for healthcare professionals and 14 for caregivers. Both guides were translated into English by a fluent speaker (MLJ) and reviewed by the research team. The questions were then validated by one pilot occupational therapist familiar with CGA and a caregiver (mother of two young children). Both participants completed the interview and subsequently provided feedback. Revisions were made to improve clarity based on this feedback. The validation of the interview guides was informed by a structured validation scale.^18^ Questions from both guides were asked. During editing, three sections were considered based on the research team's experience working with CGA in clinical settings: (1) experiences and expectations (e.g. ‘What is your impression so far of CGA as an assessment tool?’), (2) postevaluation report (e.g. ‘How could the development and use of a reference database be beneficial to your interpretations?), and (3) impact of CGA (e.g. ‘In your opinion, what is the relevance of automatically offering a gait analysis as one of the methods of initial assessment?’).

### Data collection

Each semistructured interview, lasting between 25 minutes and 45 minutes, was recorded (audio and video) using Microsoft Teams (version 2.4). The sociodemographic questionnaire was completed first. Participants were asked about their understanding of CGA, and the interviewer validated responses before proceeding to the open‐ended section. The interviewer (MLJ) has a background in CGA and kinesiology and no relationship with the participants. Interviews were completed in English or French. Participants were encouraged to ask questions and mention relevant complementary topics. To minimize potential power dynamics, particularly with the caregivers, the interviewer repeatedly stated that the study's exploratory objective was to obtain their perspective.

### Data analysis

Descriptive statistics (mean, standard deviation) were calculated for the sociodemographic data. Video interview recordings were converted into audio files and manually transcribed by MLJ and MRD. All transcripts were verified by the primary author (MLJ) during the familiarizing phase. The data were analyzed using an inductive thematic approach based on the outline guide proposed by Braun and Clarke.^19^ This approach is particularly well suited to capturing practice‐based insights and generating clinically relevant findings without imposing a priori theoretical assumptions.

The interviews were coded line by line using NVivo software (version 1.7.1, Lumivero, Denver, CO, USA) by MLJ and interjudge (MRD) validation was done for the first three. An initial code reference was created based on the questionnaire's sections and updated throughout the coding process. Other members of the research team incorporated external feedback. The research team identified and reviewed the main categories and themes to provide a final overview. To further minimize bias, an iterative process of refining and redefining codes was employed to ensure accurate representation of the data and was validated by a second researcher to mitigate subjective interpretation.

While we acknowledge that the research team's professional backgrounds may have influenced the analysis, involving multiple researchers, including members unfamiliar with CGA, likely reduced this potential bias. In addition, the researcher (MLJ) in charge of conducting the interviews and data analysis has research experience working with CGA and as a clinician working with multiple populations. Although thematic analysis was used, this study aimed to summarize participants' perspectives. Nevertheless, while informed by a constructivist perspective, the researcher's combined experience may lead to certain realities being more prominently highlighted, given the known relevance of CGA and the complexities of establishing recommendations in a clinical context. As most interviews were conducted in French, a fluent speaker team member (MLJ) translated the quotations in the results section, and the research team revised them.

## RESULTS

### Participant demographic information

Tables 1 and 2 present the characteristics of healthcare professionals and the caregivers.

**TABLE 1:** Classification of healthcare professionals (*n* = 15) in relation to clinical experience.

Characteristic	Clinical experience <10 years	Clinical experience ≥10 years	Total
Male	0	1	1
Female	6	8	14
Occupation			
Orthopedist	1	1	2
Physiatrist	3	2	5
Physical therapist	2	5	7
Clinical nurse	0	1	1

Mean age = 43 years (SD 11 years); mean clinical experience = 15 years (SD 10 years).

**TABLE 2:** Caregivers' characteristics.

Characteristics	Total, mean (SD)
Age in years	45 (5)
Completed clinical gait analysis per child	2 (1)
Age in years of children with cerebral palsy	13 (5)

The analysis highlighted two themes: (1) current and (2) desired CGA practices. Recurring topics emerged: satisfaction with CGA use and resource availability, CGA's impact, standardization, and harmonization.

### Theme 1: Current CGA practices

Every healthcare professional was familiar with and, to some extent, satisfied with current practices for quantifying gait in children with CP. They reported that CGA is often used after initial screenings (e.g. observation) in more complex cases to provide an objective perspective.

‘I have young ones with major foot deformations, and there is not much in the literature about their gait … we realize that it is super heterogeneous, so it is interesting to go case by case’ (Healthcare professional 11, physical therapist).

Most healthcare professionals highlighted that CGA helped validate their clinical impressions and explore alternatives when previous treatments were ineffective. All participants recognized CGA's personalized approach as beneficial in prioritizing both patient and family goals. Healthcare professionals also emphasized the value of combining observations with CGA to better understand a patient's condition and tailor interventions.

‘Personally … It should be a mandatory pre‐operative assessment to ensure we do what we should’ (Healthcare professional 10, orthopedist).

Healthcare professionals also highlighted that CGA can help avoid unnecessary interventions and minimize the burden on families. Caregivers echoed this satisfaction, feeling reassured when decisions were based on CGA reports and appreciating the guidance provided.

‘I was happy that she (healthcare professional) was … being very transparent about wanting to have additional information to make a better‐informed decision. So I appreciated that’ (Caregiver 7).

However, a few reported experiencing abandonment due to delays and expressed concerns about insufficient follow‐up.

‘I guess an expectation that the information would be shared more quickly between the centers’ (Caregiver 7).

Regarding available financial and human resources, which vary across centers, healthcare professionals reported limited access to CGA, with more limitations noted in centers without a laboratory coordinator. Healthcare professionals highlighted that limited staffing and/or funding are causing delays, resulting in unmet clinical needs, such as care prioritization.

‘Better not be in a hurry to have it or to decide, because otherwise, we will go towards something else … not sufficiently automated or well‐established for it to be a smooth trajectory’ (Healthcare professional 2, physiatrist).

Some caregivers noted this lack of structure and wished their child had access to CGA sooner.

‘I was disappointed that it isn't just readily available for everybody whenever they need it’ (Caregiver 7).

‘I think any child with any type of cerebral palsy of any sort would benefit from the gait laboratory for sure’ (Caregiver 1).

Based on healthcare professionals' testimonies, the limited resources undermine care quality and create referral barriers, such as challenging communication between CGA laboratories and medical teams and unclear eligibility criteria for healthcare professionals working only in clinics.

‘I find that there are still big gaps between the clinic and research … we are less likely if we are not already involved in a research project with them to use it … I think there is a professional barrier that makes us less likely to go’ (Healthcare professional 13, physical therapist).

Healthcare professionals from two centers reported attempting to structure postCGA trajectories with the support of their coordinators, research team, or laboratory. Their strategies included adding review meetings and formalizing medical recommendations through a signed document. However, healthcare professionals stated that implementing these practices in clinical settings was not always feasible, leading healthcare professionals to have to interpret reports independently.

Some healthcare professionals viewed themselves as CGA experts because of completing a gait course, a fellowship, or equivalent training.

‘It takes a certain level of knowledge to be able to interpret the graphs and detect interactions between the curves’ (Healthcare professional 1, physical therapist).

Nonetheless, some reported challenges, particularly during the collection and interpretation of CGA data. Most healthcare professionals highlighted that complex clinical profiles often hinder CGA referrals because of CP's heterogeneity in physical and mental abilities.

‘We realize that doing a more detailed analysis won't be possible with that patient because he wears out … He's unable or doesn't understand the instructions for various reasons’ (Healthcare professional 5, orthopedist).

Experienced healthcare professionals advocate for basic in‐house training for those applying treatment plans.

‘You cannot just take the CGA's results. It doesn't provide a clear prescription or answer. You still must use the clinical data and understand … what is behind the vision of the gait lab’ (Healthcare professional 12, physiatrist).

Healthcare professionals from all centers have mentioned the value of an interdisciplinary team, as this allowed for results‐based approaches to reduce trial and error when correctly mastered. Healthcare professionals indicated that working with a team enhances interpretation of the results by saving time.

‘I think it's a great gateway for multidisciplinary exchanges … I think it complements the rest … to identify cases better and optimize interventions’ (Healthcare professional 15, physical therapist).

Healthcare professionals reported that CGA allows for better knowledge transfer into their practice, positively impacting care management.

‘It (CGA) makes me realize the real limit of my eyes, which is the limit of what I can observe on my own … It allows me to be more critical … There are a lot of additional complexities I hadn't noticed before, so I think that's what helped me’ (Healthcare professional 11, physical therapist).

This was also highlighted by healthcare professionals from centers with limited access who stated the importance of implementing such a team. Overall, most healthcare professionals mentioned that CGA was complementary to their practice and could ensure long‐term collaboration with caregivers, as highlighted by the latter:

‘Some (healthcare professionals) were very skilled in communication, others less, but surely always competent … they look at him then they come up sometimes with an ultra‐fast diagnosis … this kind of tool (CGA), I see it as something more … the gait lab is much more concrete’ (Caregiver 3).

But as CGA is not the standard of care in most centers, with only research and no dedicated clinical team, this tamed the enthusiasm.

### Theme 2: Desired CGA practices

A consensus emerged among healthcare professionals and caregivers wishing to increase the number of referrals annually. Nonetheless, several acknowledged barriers. Healthcare professionals from all centers noted that CGA's impacts are predominantly positive despite non‐optimized management.

‘I think we are still working on reading, interpreting, and being as efficient as possible. I don't think we've reached our absolute maximum’ (Healthcare professional 2, physiatrist).

As for caregivers, they have mostly noted the importance to have more access to CGA as this could have a significant positive impact on their children's life.

‘I feel like the assessment is a tool to help the surgeons make a decision about improving the quality of like and the day‐to‐day activities … it sounds like it'll be worth it, based on what she's explained to me’ (Caregiver 7).

All healthcare professionals highlighted the need for a more organized approach by introducing standardized documents (e.g. clinical guides, decisional algorithms, and protocols). They advocated for a structured system to ensure consistency regardless of who collects, interprets, or analyzes the data. Healthcare professionals also viewed harmonizing CGA's methods across the centers as an impactful improvement.

‘I really think it could be extremely interesting that everyone uses the same tools every time. It would be easier, even when patients change centers, to have a standard evaluation’ (Healthcare professional 15, physiatrist).

Healthcare professionals viewed a data management system as essential for comparing cases, drawing evidence‐based conclusions, and enhancing their knowledge on complex or less‐documented conditions with the addition of a common standard. Healthcare professionals from two centers reported attempting smaller‐scale data consolidation but lacked sustainable solutions for their limited financial and/or human resources. This collaborative perspective was also mentioned, creating possible advantages (e.g. care reviews and multicenter research) since each center's specialties would be identified.

‘There are major disparities from one model to another, so by harmonizing, at least in Quebec, we can agree on the same way of doing it. It can help us precisely to develop data on certain types of pathologies and certain types of intervention …’ (Healthcare professional 3, physiatrist).

Healthcare professionals expressed mixed opinions on providing customized reports to caregivers. While viewed as a valuable tool for communication and advocacy, the additional workload of summarizing findings and recommendations for each patient, along with a follow‐up meeting, was a concern. Caregivers also expressed contrasting perspectives: some wanted as much information as possible, while others found it overwhelming because of limited knowledge.

## DISCUSSION

Technological advances have made CGA a valuable tool for acquiring quantitative data and enhancing treatment planning. The existing literature suggests that the integration of CGA into clinical settings has made minimal progress.^6^, ^20^ Support from staff and experts is crucial to ensure the long‐term adoption of CGA.^15^, ^21^ Furthermore, users' perceptions of utility and convenience are essential to understanding the reasons behind adopting or rejecting a technology.^21^ Participants were identified as key facilitators since they recognized CGA's role in caring for ambulatory children with CP and understood the benefits of an interdisciplinary, patient‐centered approach.

The primary barriers identified were financial and human resources, which are often limited in Quebec's current healthcare system. Other structural constraints, such as limited clinician expertise in interpreting complex CGA outputs, insufficient integration into clinical workflows, and lack of standardized reference data, also reduce the effective use of CGA. Together, these factors help explain why persistent barriers continue despite awareness of CGA's benefits. Importantly, the presence of dedicated coordinators or champion roles was highlighted as a potential enabler of consistent CGA use, fostering collaboration among healthcare professionals, supporting interpretation, and ensuring timely communication with caregivers.

### Impacts on healthcare professionals

A recent study suggests that the presence or absence of barriers influences how healthcare professionals use CGA, a finding echoed by participants in this study.^15^ The current process of initiating CGA management, interpreting results, and bridging clinical information to the caregivers is not based on any clinical guidelines specifically developed for this assessment tool. In centers without a dedicated clinical or laboratory team, willingness to use CGA is tempered by the added workload and the complexity of interpretation. Some sites have attempted to establish structured care trajectories, but they have been unsuccessful over time, particularly because of the lack of continuous allocation of both human and financial resources.

The lack of structure and resources seems to reduce CGA's perceived accessibility. Orthopedists and physiatrists identified complex cases as ideal candidates for CGA but recognizing them depends on individual expertise. Across centers, physical therapists reported that poorly defined selection criteria limit their ability to identify suitable patients, which may reduce their willingness to use CGA.^15^ Other factors, including long wait times to schedule the CGA, delays in receiving reports, high costs, and limited protected time to review and discuss CGA results, further constrain utilization. Centers with dedicated coordinators or champion roles were perceived as more successful in bridging these gaps, supporting interdisciplinary collaboration, standardizing procedures, and ensuring that CGA results effectively inform care pathways.

### Impacts on caregivers

Caregivers expressed mixed perceptions regarding the communication and structural support surrounding CGA, with some reporting minimal guidance throughout the process. A strong and sustained connection between healthcare professionals and caregivers was identified as essential, as CP management requires a long‐term partnership. This aligns with the people‐centered framework endorsed by the World Health Organization, which emphasizes openness, transparency, and shared decision‐making.^22^ However, caregivers' preferences for information sharing vary based on their knowledge, experience, and anxiety levels, highlighting the ‘double‐edged nature of information’ within the complex dynamics of pediatric care.^23^

To support informed decision‐making, healthcare professionals must therefore be able to tailor how CGA findings and recommendations are communicated to caregivers. Participants emphasized that the complexity of CGA results requires adequate training to ensure that information is both accurate and accessible, yet no specific communication or interpretation curriculum currently exists. Depending on funding, staff availability, and targeted services, each center should implement a team adapted to its context to support both CGA interpretation and caregiver communication, without requiring a fixed disciplinary ratio.^24^ Healthcare professionals believed that clear guidelines and training would reduce ambiguity, contribute to a more structured management, and facilitate sustained caregiver engagement, recognizing caregivers' central role in final treatment decisions.

### Limitations

Some limitations should be considered when interpreting the results, including the relatively small number of participants with current or past CGA experience. Nevertheless, recruitment continued until data saturation was achieved or full representation across participant categories and sites was reached. Results are also context dependent and tied to each site's environment, which may limit their transferability. However, other studies have reported complementary findings.^14^, ^16^, ^21^

### Conclusion

This study identified key barriers and facilitators influencing the adoption of CGA in pediatric rehabilitation centers in Quebec, with implications for similar healthcare systems. Findings underscore the importance of assessing local conditions before introducing new strategies. Healthcare professionals expressed interest in changes that would create lasting impacts in clinical practices. They emphasized addressing disparities through structural improvements, institutional support, interdisciplinary and intercenter collaboration, and interpretation training. Caregivers viewed CGA as helpful in guiding decision‐making by making results more tangible. Future research should explore tailored implementation strategies, broader harmonization efforts, and the long‐term impact of CGA‐informed interventions on patient outcomes.

## CONFLICT OF INTEREST STATEMENT

The authors declare no conflicts of interest.

## Supporting information


**Appendix S1:** Interview guide for health professionals and caregivers – French.

**Appendix S2:** Interview guide for health professionals and caregivers – English.

## Data Availability

The data that support the findings of this study are available from the corresponding author upon reasonable request.

## References

[1] Jackman M , Sakzewski L , Morgan C , Boyd RN , Brennan SE , Langdon K , et al. Interventions to improve physical function for children and young people with cerebral palsy: international clinical practice guideline. Dev Med Child Neurol. 2022;64(5):536–49. 34549424 10.1111/dmcn.15055

[2] Dan B , Rosenbaum P , Carr L , Gough M , Coughlan J , Nweke N . Updated description of cerebral palsy. Dev Med Child Neurol. 2026;68:465–476. 41612151 10.1111/dmcn.70149

[3] Novak I , Morgan C , Fahey M , Finch‐Edmondson M , Galea C , Hines A , et al. State of the Evidence Traffic Lights 2019: Systematic Review of Interventions for Preventing and Treating Children with Cerebral Palsy. Curr Neurol Neurosci Rep. 2020;20(2):3. 32086598 10.1007/s11910-020-1022-z PMC7035308

[4] Ko J , Kim M . Reliability and responsiveness of the gross motor function measure‐88 in children with cerebral palsy. Phys Ther. 2013 Mar;93(3):393–400. 23139425 10.2522/ptj.20110374

[5] Russell DJ , Rosenbaum PL , Cadman DT , Gowland C , Hardy S , Jarvis S . The Gross Motor Function Measure: A Means to Evaluate the Effects of Physical Therapy. Dev Med Child Neurol. 1989;31(3):341–52. 2753238 10.1111/j.1469-8749.1989.tb04003.x

[6] Wren TAL , Tucker CA , Rethlefsen SA , Gorton GE , Õunpuu S . Clinical efficacy of instrumented gait analysis: Systematic review 2020 update. Gait Posture. 2020 July 1;80:274–9. 32563727 10.1016/j.gaitpost.2020.05.031

[7] Benedetti MG , Beghi E , De Tanti A , Cappozzo A , Basaglia N , Cutti AG , et al. SIAMOC position paper on gait analysis in clinical practice: General requirements, methods and appropriateness. Results of an Italian consensus conference. Gait Posture. 2017 Oct 1;58:252–60. 28825997 10.1016/j.gaitpost.2017.08.003

[8] States RA , Krzak JJ , Salem Y , Godwin EM , Bodkin AW , McMulkin ML . Instrumented gait analysis for management of gait disorders in children with cerebral palsy: A scoping review. Gait Posture. 2021 Oct;90:1–8. 34358847 10.1016/j.gaitpost.2021.07.009

[9] Khouri N , Desailly E . Contribution of clinical gait analysis to single‐event multi‐level surgery in children with cerebral palsy. Orthop Traumatol Surg Res. 2017 Feb;103(1):S105–11. 27988239 10.1016/j.otsr.2016.11.004

[10] Armand S , Decoulon G , Bonnefoy‐Mazure A . Gait analysis in children with cerebral palsy. EFORT Open Rev. 2016 Dec;1(12):448–60. 28698802 10.1302/2058-5241.1.000052 PMC5489760

[11] Lennon N , Church C , Salazar‐Torres JJ , Kalisperis F , Miller F , Howard JJ . Changes in Motor Function in a Child with Cerebral Palsy Following Multiple Botulinum Toxin Injections: A Case Report. Children. 2025 June 12;12(6):761. 40564719 10.3390/children12060761 PMC12191172

[12] De Freitas Guardini KM , Kawamura CM , Lopes JAF , Fujino MH , Blumetti FC , De Morais Filho MC . Factors related to better outcomes after single‐event multilevel surgery (SEMLS) in patients with cerebral palsy. Gait Posture. 2021 May;86:260–5. 33813186 10.1016/j.gaitpost.2021.03.032

[13] Min JJ , Kwon SS , Sung KH , Lee KM , Chung CY , Park MS . Factors affecting GDI improvement after single event multilevel surgery in patients with cerebral palsy. Gait Posture. 2020 July;80:101–5. 32497978 10.1016/j.gaitpost.2020.05.033

[14] Hebda‐Boon A , Tan XL , Tillmann R , Shortland AP , Firth GB , Morrissey D . The impact of instrumented gait analysis on decision‐making in the interprofessional management of cerebral palsy: A scoping review. Eur J Paediatr Neurol. 2023 Jan 1;42:60–70. 36563467 10.1016/j.ejpn.2022.11.007

[15] Hebda‐Boon A , Zhang B , Amankwah A , Shortland AP , Morrissey D . Clinicians' Experiences of Instrumented Gait Analysis in Management of Patients with Cerebral Palsy: A Qualitative Study. Phys Occup Ther Pediatr. 2022 July 4;42(4):403–15. 35168473 10.1080/01942638.2022.2037808

[16] Armand S , Sawacha Z , Goudriaan M , Horsak B , van der Krogt M , Huenaerts C , et al. Current practices in clinical gait analysis in Europe: A comprehensive survey‐based study from the European society for movement analysis in adults and children (ESMAC) standard initiative. Gait Posture. 2024 June 1;111:65–74. 38653178 10.1016/j.gaitpost.2024.04.014

[17] Palinkas LA , Horwitz SM , Green CA , Wisdom JP , Duan N , Hoagwood K . Purposeful sampling for qualitative data collection and analysis in mixed method implementation research. Adm Policy Ment Health. 2015 Sept;42(5):533–44. 24193818 10.1007/s10488-013-0528-y PMC4012002

[18] Sánchez‐Guardiola Paredes C , Aguaded Ramírez EM , Rodríguez‐Sabiote C . Content Validation of a Semi‐Structured Interview to Analyze the Management of Suffering. Int J Environ Res Public Health. 2021 Oct 29;18(21):11393. 34769919 10.3390/ijerph182111393 PMC8583067

[19] Braun V , Clarke V . Using thematic analysis in psychology. Qual Res Psychol. 2006 Jan;3(2):77–101.

[20] Stebbins J , Harrington M , Stewart C . Clinical gait analysis 1973‐2023: Evaluating progress to guide the future. J Biomech. 2023 Nov;160:111827. 37844470 10.1016/j.jbiomech.2023.111827

[21] Sharma Y , Cheung L , Patterson KK , Iaboni A . Factors influencing the clinical adoption of quantitative gait analysis technology with a focus on clinical efficacy and clinician perspectives: A scoping review. Gait Posture. 2024 Feb;108:228–42. 38134709 10.1016/j.gaitpost.2023.12.003

[22] World Health Assembly 69 . Framework on integrated, people‐centred health services: report by the Secretariat. 2016 [cited 2025 Feb 25]; Available from: https://iris.who.int/handle/10665/252698

[23] Hummelinck A , Pollock K . Parents' information needs about the treatment of their chronically ill child: A qualitative study. Patient Educ Couns. 2006 Aug 1;62(2):228–34. 16139981 10.1016/j.pec.2005.07.006

[24] Marín J , Blanco T , Marín JJ , Moreno A , Martitegui E , Aragüés JC . Integrating a gait analysis test in hospital rehabilitation: A service design approach. PLOS ONE. 2019 Oct 30;14(10):e0224409. 31665158 10.1371/journal.pone.0224409 PMC6821402

