# Supplementary material for: Clinical gait analysis use in management of children with cerebral palsy: Qualitative study
Source: Dev Med Child Neurol. 2026 Feb 21;68(10):1390–7. doi: 10.1111/dmcn.70216 (PMC13545476; doi:10.1111/dmcn.70216)
Supplement: Supplementary file 1 [file DMCN-68-1390-s001.docx]

**Health professionals**

Sociodemographic Questionnaire

1. How old are you?
2. What is your sex?
3. Which gender do you associate with?

4. What is your highest level of education completed?

5. What is your occupation?

7. In which center are you currently working, and in which program?

8. How many years of experience do you have, and how many with the CP population?

**Interview guide –Health professionals Version**

**Theme 1 : gait analysis / experiences and expectations**

**Theme 1.1 Current methods and knowledge about gait evaluation**

1. What are the methods you are using to evaluate gait in your practice?
2. What are the reasons why you use those methods??
   a. In your opinion, what are the benefits and limits of your current methods?
3. What is your level of satisfaction with the use of those methods in your center?
4. What is your knowledge about clinical gait analysis? (gait lab)

*Quick presentation of CGA*

**Theme 1.2 Expectations of CGA**

5. What is your impression so far of CGA as an evaluation tool?
6. Do you have any suggestions to improve your gait evaluations?
7. What are your expectations once the CGA is completed?
8. According to you, À votre avis, what is the relevance of automatically offering CGA as one of the initial assessment methods during the management of a child with CP?

**Theme 2 CGA Report**

9. According to you, what are the skills required for reading and understanding a CGA report?
10. 10.	In light of the observed limitations of your current assessments, how could the use of a detailed CGA report meet your needs?
11. What are the essential elements that should be included in a CGA report?
12. What are the variables that you would find necessary to include in a CGA report?
13. In your opinion, what is the relevance of developing a simplified CGA report that could be sent to caregivers or families?
14. How could the development and use of a database as a reference be beneficial for your interpretations?

**Theme 3 CGA impact**

15. How could the use of an AQM influence your clinical or surgical practice?
    a. What would be the impact of using a CGA in the short, medium and long term?
16. According to you, to what extent could CGA help promote multidisciplinary exchanges?
17. Due to the existence of 3 centers that carry out pediatric CGA in Quebec, how do you envision the harmonization of practices between the different centers allowing for data sharing and facilitating access to this service?
18. Do you have any other suggestions or recommendations that you would like to share with us?

**Caregivers – English**

Sociodemographic questionnaire – parents

1. How old are you?
2. Which sex are you?
3. Which gender do you associated with?
4. What is your highest level of education completed?
5. What is your occupation?
6. What is your family's status?
7. How many children do you have?

Child

1. How old is your child?
2. Which sex is your child?
3. Which gender does your child associate with?
4. What grade level your child is currently completing or has completed in the last school year?
5. What is your child’s diasgnosis?
6. Who is the primary caregiver for your child?

**Theme 1 : gait analysis / experiences and expectations**

1.1 **Current knowledge of gait analysis**

1. Did you child ever got a gait analysis done in the past? If so, what is your satisfaction level about the methodologies used?
2. Do you have any knowledge about gait analysis? If so, on what extent?
3. Did your child ever had a gait analysis done in a lab before?
4. In your opinion, how relevant is to analyze your child’s gait?
   a. What are the reasons why you would authorize your child to pass that kind of assessment?
   1.2 **expectations of gait analysis**
5. What are your expectations once the gait analysis has been done?
6. What are your concerns and/or questions about the use of a gait analysis with your child?
7. What is your perception of the gait analysis in the management of your child’s treatment plan and in your child’s daily activities?

**Theme 2: Gait analysis report**

8. Given your expectations and concerns, what is the relevance of sending you the main results of your child’s gait analysis?
9. In your opinion, do you considerer useful if the gait analysis report is sent to the people working with your child on a regular basis (at school, community-based, clinical settings, etc.)? Explain.
10. According to you, how would it be beneficial or not to ask the professionals on file to explain the results to the family?
11. In your point of view, why would it be interesting or not to compare your child’s gait analysis results to the ones from a neurotypical child’s?

**Theme 3: the impacts of a gait analysis**

12. According to you, in what way does your child’s gait analysis would improve the efficiency of the current interventions both medically and socially?
13. In your opinion, what is the relevance of automatically offering a gait analysis as one of the methods of initial assessment?
14. Do you have any other suggestions or recommendations you would like to share with us?
