# Supplementary material for: Clinical gait analysis use in management of children with cerebral palsy: Qualitative study
Source: Dev Med Child Neurol. 2026 Feb 21;68(10):1390–7. doi: 10.1111/dmcn.70216 (PMC13545476; doi:10.1111/dmcn.70216)
Supplement: Supplementary file 2 [file DMCN-68-1390-s002.docx]

**VERSION CLINICIENS**

Questionnaire sociodémographique

1. Quel âge avez-vous ?
2. Quel est votre sexe ?
   - Homme
   - Femme
   - Préfère ne pas répondre
3. Quel est votre genre ?
   - Féminin
   - Masculin
   - Non binaire
   - Préfère ne pas répondre

4. Quel est votre plus haut niveau de scolarité complété ?

- Collégial
- Baccalauréat
- Maîtrise
- Doctorat
- Autre (spécifiez s’il vous plaît)

6. Quelle est votre profession?

7. Dans quelle institution travaillez-vous et dans quel programme?

8. Combien d’années d’expérience avez-vous et combien avec cette clientèle?

**Guide d’entrevue – VERSION CLINICIENS**

**Thématique 1 Analyse quantifiée de la marche / expériences et attentes**

**Thématique 1.1 Méthode et connaissance actuelle sur l’évaluation de la marche**

1. Quelles sont les méthodes que vous utilisez afin d’évaluer la marche dans votre pratique?
2. Quelles sont les raisons pour lesquelles vous utilisez cette méthode?
   a. Selon vous, quels sont les avantages et les inconvénients de votre méthode d’évaluation actuelle?
3. Quel est votre niveau de satisfaction quant à l’utilisation de la méthode utilisée dans votre centre?
4. Quelles sont vos connaissances sur l’analyse quantifiée de la marche ?

*Présentation rapide de l’analyse quantifiée de la marche avec une diapo*

**Thématique 1.2 Attentes sur l’analyse quantifiée de la marche**

5. Quelle est votre impression jusqu’à présent de l’analyse quantifiée de la marche en tant qu’outil d’évaluation?
6. Est-ce que vous avez des recommandations à suggérer pour améliorer vos évaluations de la marche?
7. Quelles sont vos attentes à la suite de l’utilisation d’une analyse quantifiée de la marche ?
8. À votre avis, quelle est la pertinence d’offrir automatiquement l’AQM comme l’une des méthodes d’évaluation initiale lors de la prise en charge d’un enfant ?

**Thématique 2 Rapport d’Analyse Quantifiée de la Marche (AQM)**

9. Selon vous, quelles sont les compétences requises pour la lecture et la compréhension d’un rapport AQM?
10. À la lumière des limitations observées de vos évaluations actuelles, en quoi l’utilisation d’un rapport détaillé de l’AQM pourrait-elle permettre de répondre à vos besoins?
11. Quels sont les éléments importants qui devraient être inclus dans un rapport d’AQM?
12. Quelles sont les variables que vous trouveriez nécessaires à inclure dans un rapport d’AQM?
13. Selon vous, quelle est la pertinence de développer un rapport simplifié d’AQM qui pourrait être transmis aux personnels aidants ou aux familles?
14. Comment le développement et l’utilisation d’une base de données comme référence pourraient-ils être bénéfiques pour vos interprétations?

**Thématique 3 Retombées d’une AQM**

15. Par quel moyen l’utilisation d’une AQM pourrait-elle influencer votre pratique clinique ou chirurgicale?
    a. Quelles seraient les retombées de l’utilisation d’une AQM à court, moyen et long terme?
16. Selon vous, dans quelle mesure l’AQM pourrait-elle contribuer à favoriser les échanges multidisciplinaires?
17. En raison de l’existence de 3 centres qui réalisent l’AQM pédiatrique au Québec, comment envisagez-vous l’harmonisation des pratiques entre les différents centres permettant le partage des données et faciliter l’accès à ce service?
18. Avez-vous d’autres suggestions ou recommandations que vous souhaiteriez partager avec nous ?

**VERSION PARENTS**

Questionnaire sociodémographique - parents

1. Quel âge avez-vous ?
2. Quel est votre sexe ?
   - Homme
   - Femme
   - Préfère ne pas répondre
3. Quel est votre genre ?
   - Féminin
   - Masculin
   - Non binaire
   - Préfère ne pas répondre
4. Quel est votre plus haut niveau de scolarité complété ?
   - Secondaire
   - Collégial
   - Baccalauréat
   - Maîtrise
   - Doctorat
   - Autre (spécifiez s’il vous plaît)
5. Quelle est votre profession?
6. Quel est votre statut familial?
   - Couple avec enfant(s)
   - Couple avec enfant(s) formant une famille reconstituée
   - Divorcé à charge partielle
   - Famille monoparentale
   - Autre
7. Combien d’enfants avez-vous?

Questionnaire sociodémographique – enfant

1. Quel âge a votre enfant?
2. Quel est son sexe?
   - Homme
   - Femme
   - Préfère ne pas répondre
3. Quel est son genre? (S’il diffère de celui assigné à la naissance)
   - Féminin
   - Masculin
   - Non binaire
   - Préfère ne pas répondre
4. Quel niveau scolaire votre enfant complète actuellement ou a complété au courant de la dernière année scolaire ?
5. Quel est le diagnostic de votre enfant ?
6. Qui est le principal intervenant auprès de votre enfant ?

**Guide d’entrevue – VERSION PARENTS**

**Thématique 1 Analyse quantifiée de la marche (AQM) / expériences et attentes**

**Thématique 1.1 connaissance actuelle sur l’évaluation de la marche**

1. Est-ce que votre enfant a déjà réalisé une évaluation de la marche par le passé? Si oui, quel est votre niveau de satisfaction quant à la méthode utilisée?
2. Quelles sont vos connaissances sur l’analyse quantifiée de la marche?
3. Votre enfant a-t-il déjà bénéficié d’une analyse quantifiée de la marche en laboratoire?
4. Selon vous, quelle est la pertinence de l’analyse quantifiée de la marche chez votre enfant?
   a. Pour quelle(s) raison(s) acceptez-vous que votre enfant réalise ce genre d’évaluation?

**Thématique 1.2 Attentes sur l’analyse quantifiée de la marche**

5. Quelles sont vos attentes à la suite de l’utilisation d’une analyse quantifiée de la marche ?
6. Quels sont vos inquiétudes et/ou questionnements sur l’utilisation de l’analyse quantifiée de la marche auprès de votre enfant?
7. Selon vous, quelle est votre perception de l’analyse quantifiée de la marche pour la prise en charge de votre enfant ainsi que la pratique des activités de la vie quotidienne?

**Thématique 2 Rapport d’AQM**

8. Compte tenu de vos attentes ainsi que de vos inquiétudes, quelle est la pertinence de vous transmettre les principaux résultats de l’analyse quantifiée de la marche ?
9. À votre avis, considéreriez-vous comme avantageux le fait de transmettre le rapport d’AQM aux personnes travaillant auprès de votre enfant (milieu scolaire, communautaire, clinique, etc.)? Justifiez.
10. Selon vous, en quoi une vulgarisation des résultats de la part des professionnels présents au dossier serait-elle bénéfique?
11. À vos yeux, pourquoi serait-il intéressant ou non de comparer les données obtenues aux résultats d’AQM d’enfants à développement typique?

**Thématique 3 Retombées d’une AQM**

12. Selon vous, en quoi l’analyse quantifiée de la marche chez votre enfant bonifierait-elle l’efficacité des interventions déjà en cours autant sur le plan médical que sur le plan social?
13. ~~En quoi la transmission des résultats aux intervenants sociaux ainsi qu’au personnel intervenant auprès de votre enfant en milieux scolaires serait-elle pertinente?~~
14. À votre avis, quelle est la pertinence d’offrir automatiquement l’AQM comme l’une des méthodes d’évaluation initiale lors de la prise en charge d’un enfant ?
15. Avez-vous d’autres suggestions ou recommandations que vous souhaiteriez partager avec nous ?
